# 312. Strain Engraftment and Gene Transfer in a Fecal Microbiota Transplantation Cohort

**DOI:** 10.1093/ofid/ofad500.384

**Published:** 2023-11-27

**Authors:** Catherine Brink, Roth Conrad, Ahmed Babiker, Janet Hatt, Danielle Barrios-Steed, Colleen S Kraft, Michael H Woodworth, Konstantinos Konstantinidis

**Affiliations:** Georgia Institute of Technology, Atlanta, Georgia; Georgia Institute of Technology, Atlanta, Georgia; Emory University School of Medicine, Atlanta, GA; Georgia Institute of Technology, Atlanta, Georgia; Emory University School of Medicine, Atlanta, GA; Emory University, Atlanta, GA; Emory University, Atlanta, GA; Georgia Institute of Technology, Atlanta, Georgia

## Abstract

**Background:**

Fecal microbiota transplantation (FMT) is a procedure that has gained popularity due to the high efficacy in treating recurrent *Clostridioides difficile* infections (RCDI) and other conditions associated with a dysbiotic gut microbiome. Despite the widespread acceptance of the technique, repeating its success in RCDI treatment in other diseases has been difficult due to the individual-level variation in disease effects on the gut and the emphasis on taxonomic, rather than functional, microbiome analyses. We present a metagenomic analysis of the use of FMT to treat renal transplant patients infected with multidrug-resistant organisms (MDROs).

**Methods:**

DNA was extracted from longitudinally collected participant stool samples before and after FMT. Reads were assembled and binned into metagenome assembled genomes (MAGs), which were then mapped back to the reads to calculate relative abundance, breadth and sequencing depth in each patient sample. This allowed us to assess the species-level changes in the composition of patient microbiomes after FMT treatment.

**Results:**

We identified four MAGs, representing close relatives of A*kkermansia muciniphila*, *Dakarella massiliensis*, *Mesosutterella multiformis* and *Waltera intestinalis,* that showed a pattern of engraftment (absent or undetectable before FMT, but present for at least two consecutive timepoints after treatment) in FMT-treated patients. We also observed clear evidence of a donor MAG closely related to *Faecalibacterium gallinarum* replacing patient MAGs identified as *Faecalibacterium hattori*, revealing a potential mechanism of successful FMT treatment though a resistant strain in the patient microbiome likely being outcompeted by a drug-susceptible strain of the same species from the donor.

**Conclusion:**

Herein we elucidated the taxonomic changes that take place in renal transplant patient gut microbiomes with reduced MDRO colonisation in response to FMT treatment. Future efforts will aim to identify the shifts in the functional gene profiles of the patient microbiomes to allow for a deeper understanding of the mechanisms underlying successful FMT treatments and further the rational design of live biotherapeutic consortia to treat antibiotic-resistant colonisation.

**Disclosures:**

**Ahmed Babiker, MBBS**, Roche: Advisor/Consultant

